# Preoperative treatment with radiochemotherapy for locally advanced gastroesophageal junction cancer and unresectable locally advanced gastric cancer

**DOI:** 10.2478/raon-2014-0027

**Published:** 2015-03-25

**Authors:** Ivica Ratosa, Irena Oblak, Franc Anderluh, Vaneja Velenik, Jasna But-Hadzic, Ajra Secerov Ermenc, Ana Jeromen

**Affiliations:** Department of Radiotherapy, Institute of Oncology Ljubljana, Ljubljana, Slovenia

**Keywords:** unresectable gastric cancer, gastroesophageal junction cancer, preoperative radiochemotherapy, surgery, toxicity

## Abstract

**Background.:**

To purpose of the study was to analyze the results of preoperative radiochemotherapy in patients with unresectable gastric or locoregionally advanced gastroesophageal junction (GEJ) cancer treated at a single institution.

**Patients and methods.:**

Between 1/2004 and 6/2012, 90 patients with locoregionally advanced GEJ or unresectable gastric cancer were treated with preoperative radiochemotherapy at the Institute of Oncology Ljubljana. Planned treatment schedule consisted of induction chemotherapy with 5-fluorouracil and cisplatin, followed by concomitant radiochemotherapy four weeks later. Three-dimensional conformal external beam radiotherapy was delivered by dual energy (6 and 15 MV) linear accelerator in 25 daily fractions of 1.8 Gy in 5 weeks with two additional cycles of chemotherapy repeated every 28 days. Surgery was performed 4–6 weeks after completing radiochemotherapy. Following the surgery, multidisciplinary advisory team reassessed patients for the need of adjuvant chemotherapy. The primary endpoints were histopathological R0 resection rate and pathological response rate. The secondary endpoints were toxicity of preoperative radiochemotherapy and survival.

**Results.:**

Treatment with preoperative radiochemotherapy was completed according to the protocol in 84 of 90 patients (93.3%). Twenty patients (22.2%) did not undergo the surgery because of the disease progression, serious comorbidity, poor performance status or still unresectable tumour. In 13 patients (14.4%) only exploration was performed because the tumour was assessed as unresectable or diffuse peritoneal carcinomatosis was established. Fifty-seven patients (63.4%) underwent surgery with the aim of complete removal of the tumour. Radical resection was achieved in 50 (55.6%) patients and the remaining seven (7.8%) patients underwent non-radical surgery (R1 in five and R2 in two patients). In this group of patients (n = 57), pathological complete response of tumour was achieved in five patients (5.6% of all treated patients or 8.8% of all operated patients). Down-staging was recorded in 49 patients (86%), in one patient (1.8%) the stage after radiochemotherapy was unchanged while in seven patients (12.3%) the pathological stage was higher than clinical, mainly due to higher pN stage. No death was recorded during preoperative radiochemotherapy. Most grade 3 and 4 toxicities were due to vomiting, nausea and bone marrow suppression (granulocytopenia). Twenty-six (45.6%) patients died due to GEJ or gastric carcinoma, one died because of septic shock following the surgery and a reason for two deaths was unknown. Twenty-eight patients (49.1%) were disease free at the time of analysis, while 29 patients (50.9%) developed the recurrence, mostly as distant metastases. At two years, locoregional control, disease-free survival, disease-specific survival and overall survival were 82.9%, 43.9%, 56.9% and 53.9%, respectively.

**Conclusions.:**

Preoperative radiochemotherapy was feasible in our group of patients and had acceptable toxicity. Majority of patients achieved down-staging, allowing greater proportion of radical resections (R0), which are essential for patients’ cure.

## Introduction

Gastric and gastroesophageal junction (GEJ) cancer are two groups of tumours with different pathogenesis, epidemiology and clinicopathological characteristics.^1^,^2^ In the past years changes in classification based mainly on anatomical localization of the tumour were made, and today GEJ cancer that arises within first 5 cm of the stomach and also extends to the oesophagus, is classified as oesophageal cancer (Siewert type I, II or III).^3^ Not all of the authors agree with new staging principles, as new classification does not represent the molecular origin of carcinoma. An appropriate interpretation of results from past therapeutic trials also became difficult, as GEJ carcinomas were formerly classified and treated as gastric carcinomas.^4^–^6^

Surgery is without doubt the main part of curative treatment of primarily advanced (≥ cT3N+) gastric and GEJ cancer. The best surgical approach for both groups is still a subject of a debate, especially regarding the extension of lymphadenectomy. Panel of experts agree that gastrectomy with D2 lymph node dissection (without resection of the pancreatic tail and without routine splenectomy) is advised as the standard approach in Europe and Asia for gastric and Siewert type III GEJ cancers, while Siewert type I and II should be treated by oesophagectomy (or by extended transhiatal gastrectomy for type II tumours, if needed) with dissection of mediastinal lymph nodes.

Although surgical techniques have been improved, local recurrence rate after complete re-section of gastric and GEJ adenocarcinoma is still high.^7^–^9^ Suboptimal surgery and high rates of distant metastases - especially to liver, bone, brain and lung - have led to search for additional therapeutic approaches. The results of INT-0116 and MAGIC trials suggest a survival benefit of postoperative radiochemotherapy or perioperative chemotherapy and subsequently they were accepted as standards of care.^10^,^11^

Recent evidence shows an important role of radiotherapy in addition to preoperative chemotherapy regimens in GEJ and in gastric cancers as well as in others gastrointestinal cancers.^12^ According to the results of the CROSS trial, in which 368 patients with T1N1 or T2-3N0-1 tumours without clinical evidence of metastatic spread (M0) were included, preoperative radiochemotherapy followed by surgery significantly improves disease-free survival (DFS) and overall survival (OS) as compared with surgery alone in patients with distant oesophageal or GEJ squamous-cell carcinoma, adenocarcinoma, or large-cell undifferentiated carcinoma. A pathological complete response (pCR) and radical resection (R0) was achieved in 29% and 92% of patients who underwent resection after radiochemotherapy, compared to the 69% of R0 resections in the group where the patients were treated with surgery only.^13^ In ACCORD07 phase III trial patients treated with preoperative chemotherapy had better survival compared to the patients treated with surgery alone (5-year OS 38% vs. 24%). Survival rates from CROSS trial where preoperative radiochemotherapy was used seem to be superior then in ACCORD07 trial where preoperative chemotherapy was used (5-year OS 47% vs. 34% in control arm).^14^ Results, published by Stahl *et al*., also pointed to a survival advantage for preoperative radiochemotherapy compared with preoperative chemotherapy in GEJ carcinomas (3-year OS rate 47.4% and 27.7%, respectively). The patients treated with preoperative radiochemo-therapy also had lower rate of non-radical resections (4.1% and 14.4%, respectively) and higher rate of pCRs (15.6% and 2%, respectively). Another interesting observation in that study is that the patients who achieved pCR had 100% 3-year OS rate, whereas in other patients the 3-year OS was 47.4%.^15^

In gastric cancer, data demonstrating survival benefits of preoperative radiochemotherapy are less clear. Only several retrospective or prospective, randomized or non-randomized trials (in general with small number of included patients), investigating the role of preoperative radiochemotherapy for gastric cancer have been published. Rates of R0 resection and pCR in resectable gastric cancer patients were as high as 70–78% and 20– 30%, respectively.^16^–^25^ Authors concluded that morbidity and mortality were not significantly higher in preoperative radiochemotherapy treatment.^15^,^26^ Valenti *et al*. randomized 72 patients with operable locally advanced gastric cancer (cT3–4/N+) in two groups. The first group was treated with preoperative chemotherapy, the second one with preoperative radiochemotherapy. They did not find differences in the incidence of complications between groups (30.9% vs. 33.3%, respectively). A major pathological response was detected in 33.3% of patients and it was more frequent in the radiochemo-therapy group (47.6% vs. 13.3%, p = 0.0024).^26^ A randomized phase II/III trial (TOPGEAR) is currently recruiting patients with resectable adenocarcinoma of the stomach or GEJ to answer whether preoperative radiochemotherapy is superior to perioperative chemotherapy alone.^27^

Arguments for using the combination of chemotherapy and radiotherapy have a biological explanation: chemotherapy - by acting cytotoxically - reduces the number of cells in tumours and makes them more susceptible to radiotherapy by inhibiting cellular repair mechanisms. Radiotherapy triggered accelerated repopulation of tumour cells reduced by chemotherapy is another example of cooperation of the two modalities. Tumour shrinkage allows enhanced reoxygenation.^28^,^29^ Intact tumour vasculature in the preoperative setting helps in better chemotherapy delivery. The aim of such treatment is also to eradicate subclinical metastatic disease and to sterilize the surgical fields - potentially reducing the risk of local tumour dissemination at resection.^19^,^21^,^30^ Preoperative radiotherapy treatment fields can be smaller and the radiation delivery itself more accurate.^21^ It seems that preoperative treatment with chemotherapy or radiochemotherapy for locally advanced gastric or GEJ cancer can be performed safely, with high compliance, and acceptable toxicity profiles and low perioperative morbidity and mortality rates.^13^,^26^

Several authors reported benefits of preoperative radiochemotherapy also in unresectable gastric cancer patients showing that tumour down-staging can be achieved, enabling radical resections, in 25–50% of these patients with consequent benefit on their survival.^31^–^40^

The purpose of this study was to analyze the effectiveness and safety of preoperative radiochemotherapy in patients with unresectable gastric and locoregionally advanced GEJ adenocarcinoma treated at the Institute of Oncology Ljubljana.

## Methods

### Patient characteristics

Patients with unresectable gastric or locoregionally advanced GEJ adenocarcinoma treated in Slovenia with preoperative radiochemotherapy between January 2004 and July 2012 were included in this retrospective study. According to surgeon’s opinion gastric tumours were estimated as unresectable by endoluminal ultrasound (EUS) and/or computer tomography (CT) imaging, and GEJ tumours were estimated as locoregionally advanced by EUS and/or CT.

Patients were presented to a multidisciplinary advisory team, consisting of a surgeon, radiation oncologist and medical oncologist and were considered for preoperative treatment if the following criteria were met: histologically confirmed unresectable gastric or locoregionally advanced GEJ adenocarcinoma, age greater than 18 years and below 80 years, no prior radiotherapy and/or chemotherapy, a performance status of 2 or lower according to World Health Organization (WHO), adequate function of major organs (including cardiac, bone marrow, renal and hepatic function) and adequate collaboration during treatment. All patients underwent a general clinical examination, blood tests, endoscopy of upper gastrointestinal tract with biopsy of the tumour and EUS and/or radiographic imaging (CT of abdomen and/or thorax) to define the extent of the disease. If there was a suspicion of distant metastases (high level of tumour markers or any suspicious lesion on CT scan), positron emission tomography-computed tomography (PET-CT) was performed.

During therapy the patients were clinically examined and referred to haematology and biochemistry blood tests once a week. The therapy-related local and systemic toxicity was assessed according to the National Cancer Institute Common Toxicity Criteria (NCI-CTC) version 4.0.^41^ The performance status of patients was determined and their body weight was measured on a weekly basis. All patients received intensive supportive care, including intensive nutrition support.

For the purpose of this study patients’ disease stage was classified using medical records, according to the new, 7^th^ edition of the AJCC cancer-staging manual.^3^ Ninety patients with stages IIIA–IV of gastric carcinoma (including Siewert III) and IIB– IV of GEJ adenocarcinoma (Siewert I and II) were included in the study (Table 1).

### Treatment

All patients were treated preoperatively at the Institute of Oncology Ljubljana. The treatment schedule consisted of induction chemotherapy (one cycle) with 5-fluorouracil (1000 mg/m^2^) in 96 h continuous infusion and cisplatin (75 mg/m^2^) in a bolus on day 2, followed by concomitant radiochemotherapy four weeks later. Concomitant radiochemotherapy included two cycles of the same type of chemotherapy repeated every 28 days. Chemotherapy administration required hospitalization for appropriate monitoring, hydration, antiemetic therapy and other supportive treatment that included also nutrition support. In case of severe therapy-related toxicity, irradiation and/or chemotherapy doses were modified and adapted to the patient’s physical condition or laboratory tests. When necessary, chemotherapy application was delayed, or radiotherapy was temporarily interrupted or terminated.

**FIGURE 1.**
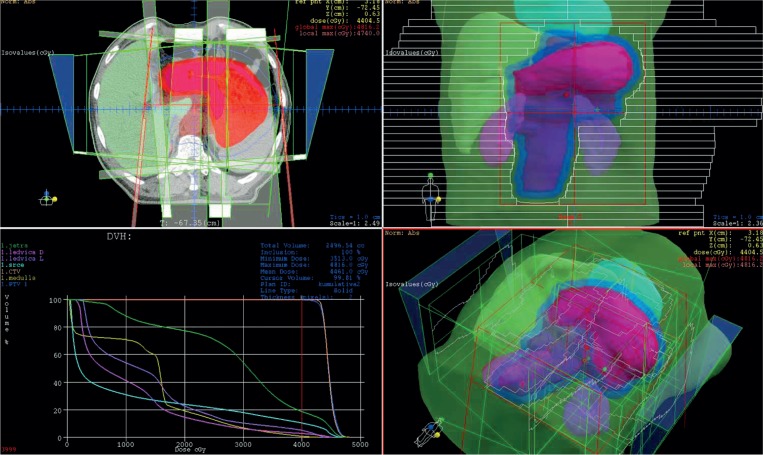
Planning target volume and dose–volume histogram for patient with locoregionally advanced gastric cancer.

Radiotherapy started at the beginning of the second cycle. Three-dimensional conformal radiotherapy was delivered by dual energy (6 and 15 MV) linear accelerator in 25 daily fractions of 1.8 Gy in 5 weeks. Planning target volume (PTV) received 45 Gy and encompassed the entire stomach or all tumour extension (if present) and draining lymph nodes (perigastric, coeliac, porta hepatis, gastroduodenal, splenic hilar, suprapancreatic, pancreaticoduodenal and paraaortic to the level of L3/L4) (Figure 1). For GEJ tumours and tumours which originated in the upper third of the stomach the upper margin of at least 3–5 cm was used in the distal oesophagus, and for distal lesions (at or near the gastroduodenal junction), a 5 cm lower margin in the part of duodenum was used. The dose was prescribed to cover the PTV with a 95% reference isodose (95% of the International Commission on Radiation Unit reference point dose). Custom shielding with multileaf collimator was applied to reduce the dose to kidneys (70% of one kidney volume < 20 Gy and 30% of second kidney volume < 20Gy), liver (30% of liver volume < 30Gy) and spinal cord (Dmax < 45 Gy). Dose-volume histogram parameters were used for plan verification regarding target coverage and normal structures sparing. Treatment was verified using a weekly portal imaging.

During the radiochemotherapy treatment, the patients were followed up on weekly basis by clinical examination and laboratory blood tests. Patients’ performance status, weight loss and toxicity profiles according to CTCAE v4.0 were registered.^41^ Surgery was performed 4–6 weeks after radiochemotherapy in two University Clinical Centers in Slovenia - Ljubljana and Maribor. Following the surgery patients were reassessed by multidisciplinary advisory team for the need of adjuvant chemotherapy.

After the treatment, patients were followed up every 3 months for 2 years and later on every 6 months until 5 years or death. The study was approved by the institutional review board committee and it was carried out according to the Declaration of Helsinki.

### Statistical analysis

Statistical analysis was performed using statistical package SPSS, version 20 (SPSS Inc., USA).

The primary endpoints were histopathological R0 resection rate and pathological response rate. The effect of preoperative radiochemotherapy on tumour down-staging was assessed by comparing the pretreatment clinical TNM stage with the postoperative pathologic TNM stage. The secondary endpoints of this study were as follows: toxicity of preoperative radiochemotherapy, early postoperative mortality and locoregional control (LRC, the event was local and/or regional recurrence), DFS (the event was local, regional or systemic recurrence), disease-specific survival (DSS, the event was death due to gastric adenocarcinoma) and OS (the event was death from any cause).

Survival data was calculated from the beginning of preoperative treatment to the November 1st 2013 (close-out date). Survival probability was calculated using Kaplan-Meier estimate^42^, and log rank test^43^ was used to evaluate the differences between individual groups of patients. Independent prognostic values of variables that appeared as statistically significant on univariate analysis were tested by multivariate Cox regression analysis model. Two-sided tests were used and the differences at p < 0.05 were considered as statistically significant.

## Results

Eighty-four patients (93.3%) completed preoperative treatment with radiochemotherapy according to the protocol. In six patients (6.7%) RT was interrupted before 45 Gy and none of those patients received the last (third) cycle of chemotherapy. In one patient treatment was interrupted at 12.6 Gy due to pulmonary abscess (as a result of communication between tumour and pulmonary system), in one patient at 14.4 Gy because of febrile neutropenia, in one patient at 33.6 Gy because of progression into the liver and in three patients at 41.4 Gy due to serious side effects of treatment and deterioration of performance status.

### Resection rate

Twenty patients (22.2%) did not undergo the surgery. In 8 patients the reason was progression of the disease with the occurrence of distant metastases during or after preoperative treatment, one patient developed ileus of small intestine and one patient died due to the rupture of colon transversum caused by direct tumour infiltration. In six patients tumour was estimated as unresectable and other four patients were not operated on due to poor performance status or serious comorbidity. In 13 patients (14.4%) only exploration was performed because the tumour was assessed as unresectable in 6 and diffuse peritoneal carcinomatosis established in 7 patients (three of them had M1 stage at diagnosis).

Fifty-seven patients (63.4%) underwent surgery with the aim of complete removal of the tumour. Except for one patient (whose surgery was performed in regional hospital) all the operations were performed in two major surgical centres in Slovenia - University Medical Centre Ljubljana and Maribor. Distal subtotal resection of the stomach was performed in 15 (16.7%) patients, total resection of the stomach in 32 (35.6%) patients, multivisceral resection in seven (7.8%) patients and transthoracic oesophageal resection in three (3.3%) patients. R0 resection was achieved in 50 (55.6%) of all patients and the remaining seven (7.8%) patients underwent non-radical surgery (R1 in five and R2 in two patients) (Table 2).

### Pathological response rate

Among the patients that underwent surgery with the aim of complete tumour removal (n = 57), pCR was achieved in five patients (5.6% of all treated patients or 8.8% of all operated patients). Down-staging was recorded in 44 patients (86%), in one patient (1.8%) the stage after radiochemotherapy was unchanged while in seven patients (12.3%) the pathological stage was higher than clinical, mainly due to higher pN stage (Table 3). We did not find any statistical differences in survival between the groups of patients with tumour/nodes down-staging versus patients with no response to preoperative treatment.

### Toxicity of preoperative radiochemotherapy

Five patients (5.6%) did not complete the treatment as planned. Reasons were lung abscess in one patient and serious side effects of the treatment (such as fatigue, neutropenic fever and serious deterioration of performance status) in other four patients. Consequently none of them received the last (third) cycle of chemotherapy.

No death was recorded during preoperative radiochemotherapy. Most grade 3 and 4 toxicities (Table 4) were due to vomiting, nausea and bone marrow suppression. In total, 58% of patients lost their weight during radiochemotherapy, but more than 10% of weight loss was seen in only 13.9% of patients.

### Outcome of the disease for patients who underwent surgery

The median follow up for all patients was 18 months (range 4–77 months), but for the subgroup of survivors the median follow up was 20 months (range 5–77 months). Twenty-six (45.6%) patients died due to GEJ or gastric carcinoma, one died because of septic shock following the surgery and a reason for two deaths was unknown. Twenty-eight patients (49.1%) were disease free, while 29 patients (50.9%) developed the recurrence: one patient (1.8%) only local, one patient (1.8%) locoregional, 24 patients (42.1%) only distant metastases and other three patients (5.4%) locoregional recurrence in combination with distant metastases. At two years, LRC, DFS, DSS and OS were 82.9%, 43.9%, 56.9% and 53.9%, respectively.

### The GEJ cancer patients (Siewert I + II) who underwent surgery

In the group of the GEJ cancer patients (Siewert I + II) 21 patients completed preoperative treatment and were operated on for the removal of the tumour. R0 resection of the tumour was achieved in 19 patients (90.5%) and the remaining 2 patients (9.6%) underwent non-radical surgery. pCR was achieved in four patients (19%). Down-staging was altogether achieved in 17 patients (81%) (Table 5).

Seven patients (33.3%) died due to GEJ carcinoma, one died because of septic shock after surgery and a reason for one death is unknown. Twelve patients (57.1%) were disease free, in one patient (4.8%) only locoregional recurrence developed and eight patients (38.1%) presented with distant metastases. At 2 years, LRC, DFS, DSS and OS were 82.3%, 47%, 56% and 50.6% respectively.

### The initially unresectable gastric cancer patients who underwent surgery

After preoperative radiochemotherapy the surgery for tumour removal was performed in 36 patients with initially unresectable cancer. R0 resection of the tumour was achieved in 31 (86.1%) patients and the remaining 5 (13.9%) patients underwent non-radical surgery. pCR of the tumour was achieved in 1 patient (2.8%) and downstaging was recorded in 32 patients (88.9%) (Table 6).

Nineteen patients (52.8%) died due to gastric cancer; in one patient (2.8%) the reason for death was unknown. Sixteen patients (44.4%) were without any signs of disease, in one patient (2.8%) only locoregional recurrence developed, sixteen patients (44.4%) developed distant metastases only and other three patients (8.4%) developed combination of locoregional recurrence and distant metastases. At 2 years, LRC, DFS, DSS and OS were 79.9%, 43.5%, 58.8% and 57.1%, respectively.

## Discussion

In the 7^th^ edition of the AJCC cancer staging manual, GEJ carcinomas have been classified as oesophageal carcinomas. However, for various reasons, some of the experts believe that their classification should remain under gastric carcinoma. Furthermore, the published literature in this area is not uniform and often deals with both localizations together. Based on various research findings GEJ cancer is currently treated with different modalities: postoperative radiochemotherapy^9^,^11^, perioperative chemotherapy with epirubicin, cisplatin and 5-fluorouracil^8^,^10^ and in few past years preoperative radiochemotherapy is being used increasingly.^14^,^15^ The current approach to the treatment of unresectable gastric cancer patients is based mainly on the preference of the oncologist and perioperative chemotherapy^10^, systemic therapy with trastuzumab for tumours with HER2 overexpression^44^ or preoperative radiochemotherapy is used.^31^–^40^ It has been shown that preoperative radiochemotherapy in GEJ carcinoma is superior to preoperative chemotherapy.^15^ In resectable gastric carcinoma the role of preoperative radiochemotherapy is not so defined, since there are currently no data to clarify the differences of both treatment modalities and results from TOPGEAR study are eagerly awaited.^27^ In patients with unresectable gastric cancer, who were offered only palliative treatment in the past, it is even more meaningful to use the combination of radiotherapy and chemotherapy in order to increase the effectiveness of the treatment and hopefully change the unresectable disease into the resectable one with R0 resection.

At our institution the same preoperative treatment for GEJ and gastric carcinomas is used. Radiation therapy is delivered daily in 1.8 Gy per fraction to the total dose of 45 Gy with concomitant chemotherapy using 5-fluorouracil and cisplatin. Surgery for both tumours follows in 4–6 weeks after the completion of preoperative radiochemo-therapy. Patients in our study had advanced disease with tumours staged as cT4 and cN+ disease in 75.6% and 93.3% of patients, respectively. Four patients with unresectable gastric carcinoma (4.4%) had localized peritoneal carcinomatosis diagnosed by laparoscopy or exploratory operation prior to treatment. Despite M1 disease multidisciplinary advisory team indicated preoperative treatment because of excellent general condition of these patients and hope for achieving radical resection. Two patients were later operated on, with R1 resection in first and only exploratory operation in the second patient. All 4 patients died due to gastric cancer within one year from completing the preoperative treatment (median: 4 months, range: 3–12 months).

Eighty-four patients (93.3%) finished preoperative treatment with radiochemotherapy according to the protocol. Only in four patients the treatment was stopped prematurely due to toxic side effects, which did not allow the continuation of therapy (neutropenic fever in one patient and serious deterioration of performance status in other three patients). No death was recorded during preoperative radiochemotherapy. Most grade 3 and 4 toxicities were due to nausea and vomiting (in 25.6% of patients) and bone marrow suppression with granulocytopenia (in 16.9% of patients). Similar toxicities were noted in other studies.^16^,^22^,^35^ Altogether 58% patients lost their weight during radiochemotherapy, but more than 10% of weight loss was seen in only 13.9% of patients, which we believe is the result of excellent team work between radiation oncologists, nutritionists and intensive supportive therapy that was provided to our patients.

Several studies have demonstrated that preoperative radiochemotherapy does not increase early postoperative mortality.^15^,^35^,^45^ In our study only one patient (1.8%) died due to septic shock early after the surgery and there were no reports of anastomotic leak or any other complications after surgery.

Only 57 patients (63.4%) in our study underwent surgery for tumour removal. In 50 patients (55.6% of all included or 87.7% of operated patients) R0 re-section was obtained. Ajani *et al*. reported that 85% of all included patients underwent surgery and in 70% R0 resection was obtained, but in this study only initially resectable, non-cT4 tumours were included.^17^

There is a general belief that all such patients should be operated in large, multidisciplinary centres, by experienced surgeons in order to achieve better treatment outcome. In our study only one patient was not operated in a large volume surgery centre, which reflects that gastric surgery in our country is centralized.

In 49 (86%) of operated patients tumour and/or nodes down-staging was achieved (when comparing clinical stage and pathological stage), which we believe is a very good result. Ajani *et al*. noted response on preoperative radiochemotherapy in 64% of patients with resectable gastric cancer who were operated and in 55% of all assessable patients.^17^ In our study pCR was achieved in only five patients (5.6% of all assessable patients) in comparison with Ajani *et al*. who reported pCR in 30% of all patients.^17^ As expected, more pCRs in our study were achieved in patients with GEJ tumour (four) comparing to only one patient with unresectable gastric cancer, as GEJ tumours were less advanced and mostly resectable. The 2-years OS of our patients was 53.9%, which is similar to the results that Ajani *et al*. reported in their study.^16^

If we consider GEJ tumours and gastric tumours separately, we can notice that patients with gastric cancer had worse 2-years LRC than patients with GEJ cancer (79.9% and 82.3%, respectively). This is somehow expected because gastric cancer patients in our study had more advanced disease considered as unresectable before the start of any treatment. On the other hand, there were no significant differences between GEJ and gastric tumours in DFS, DSS and OS at 2-years. It seems that the well-known worse outcome for GEJ tumours was not so obvious, as GEJ tumours in our study were less advanced.

The biggest limitation of our study is the retrospective data collection. For more accurate conclusions we would need longer follow-up and a larger number of enrolled patients. Maybe patients with GEJ and gastric cancer should be separated (although TOPGEAR study includes both tumour sites, without Siewert I). Furthermore, we need to develop more effective systemic drugs in order to decrease the incidence of distant metastases, which are the most common site of the disease recurrence.

In conclusion, we believe that treatment with preoperative radiochemotherapy was feasible, with acceptable toxicity, and it enabled good down-staging with even the possibility of complete pCR of the disease.

## Figures and Tables

**TABLE 1.** Patients and tumour characteristics (n = 90)

**Characteristic**		**No.**	**%**
**Gender**	Male	24	26.7
	Female	66	73.3
**PS (WHO)**	0	62	68.9
	1	26	28.9
	2	2	2.2
**Weight loss before therapy**	Yes	67	74.4
	No	23	25.6
**Primary tumour localization**	Stomach (including GEJ Siewert III)	55	61.1
**Clinical T stage**	GEJ (Siewert I+II)	35	38.9
	1	0	0
	2	2	2.2
	3	20	22.2
	4a	35	38.9
	4b	33	36.7
**Clinical N stage**	0	6	6.7
	1	16	17.8
	2	29	32.2
	3	39	43.3
**Clinical M stage**	0	86	95.6
	1*	4	4.4
**Stage grouping at presentation**	IIA	2	2.2
	IIB	5	5.6
	IIIA	12	13.3
	IIIB	11	12.3
	IIIC	56	62.2
	IV	4	4.4

GEJ = gastroesophageal junction; PS = Performance status at presentation according to WHO scoring system;

* Clinical M1 stage includes only tumours with local peritoneal carcinomatosis

**TABLE 2.** Surgery characteristics

**Surgery**		**No. of patients**	**%**
No surgery		20	22.2
Only exploratory operation		13	14.4
Subtotal gastrectomy		15	16.7
Total gastrectomy		32	35.6
Multivisceral resection		7	7.8
Transthoracic oesophagectomy		3	3.3
Type of resection	R0	50	55.6
	R1	5	5.6
	R2	2	2.2

**TABLE 3.** Pathological response rate

	**T-stage**		**N-stage**		**Overall stage**	

**Response rate**	**n**	**%**	**n**	**%**	**n**	**%**
pCR^*^	5	8.8	×	×	5	8.8
p-stage < c-stage	42	73.7	37	64.9	49	86
p-stage = c-stage	14	24.6	13	22.8	1	1.8
p-stage > c-stage	1	1.8	7	12.3	7	12.3

c = clinical; p = pathologic; pCR = pathologic complete response

**TABLE 4.** Toxicity of preoperative radiochemotherapy

**Toxicity**	**NCI grade (% of patients, n = 90)**					

	**0**	**1**	**2**	**3**	**4**	**5**
Radiomucositis	53.4	30	13.3	2.2	1.1	0
Radiodermatitis	0	0	0	0	0	0
Diarhoea	90	6.7	2.2	1.1	0	0
Dysphagia	44.4	37.8	8.9	5.6	3.3	0
Vomiting, nausea	38.8	16.7	18.9	20	5.6	0
Infection	53.4	18.9	11.1	13.3	3.3	0
Weight loss	41.7	44.4	11.1	2.8	×	×
Granulocytopenia	17.8	15.6	37.8	23.3	5.5	0
Anemia	13.3	41.1	41.1	3.3	1.1	0
Trombocytopenia	31.1	47.8	10	5.6	5.6	0

NCI = National Cancer Institute Common Toxicity Criteria for Adverse Events version 4.0 (CTCAE v4.0)^41^

**TABLE 5.** The GEJ cancer patients (Siewert I + II) who underwent surgery

**Characteristic**		**No.**	**%**
Gender	Male	17	81
	Female	4	19
Age		Median 62 years (44–80 years)	
PS (WHO)	0	17	81
	1	2	9.5
	2	2	9.5
Resectability	R0	19	90.5
	R1	1	4.8
	R2	1	4.8
Response	pCR	4	19
	pT-stage < cT-stage	14	66.7
	pT-stage = cT-stage	6	28.6
	p-Tstage > cT-stage	1	4.8
	pN-stage < cN-stage	12	57.2
	pN-stage = cN-stage	5	23.8
	pN-stage > cN-stage	4	19
	p-stage < c-stage	17	81
	p-stage = c-stage	0	0
	p-stage > c-stage	4	19

pCR = Pathological complete response; PS = Performance status according to WHO scoring system

**TABLE 6.** The unresectable gastric cancer patients who underwent surgery

**Characteristic**		**No.**	**%**
Gender	Male	25	69.4
	Female	11	30.6
Age		Median 62 years (43–78 years)	
PS (WHO)	0	28	77.8
	1	8	22.2
Resectability	R0	31	86.1
	R1	4	11.1
	R2	1	2.8
Response	pCR	1	2.8
	pT-stage < cT- stage	28	77.8
	pT-stage = cT- stage	8	22.2
	p-Tstage > cT- stage	0	0
	pN-stage < cN stage	25	69.4
	pN-stage = cN- stage	8	22.2
	pN-stage > cN stage	3	8.3
	p-stage < c-stage	32	88.9
	p-stage = c-stage	1	2.8
	p-stage > c-stage	3	8.3

pCR = Pathological complete response; PS = Performance status according to WHO scoring system
